# Computed Tomography Angiography‐Based Mapping of Septocutaneous Fibular Artery Perforators: An Anatomical Analysis

**DOI:** 10.1002/micr.70266

**Published:** 2026-07-14

**Authors:** Jannik Ketschau, Jonathan Mohr, Yannik Leonhardt, Alex Grabenhorst, Hannes Singer, Helena Kram, Nils Krautkremer, Sina Heimüller, Katharina Pippich, Herbert Stimmer, Klaus‐Dietrich Wolff, Lucas M. Ritschl

**Affiliations:** ^1^ Department of Oral and Maxillofacial Surgery TUM University Hospital Klinikum Rechts der Isar, School of Medicine and Health, Technical University of Munich Munich Germany; ^2^ Institute of Diagnostic and Interventional Radiology TUM University Hospital Klinikum Rechts der Isar, School of Medicine and Health, Technical University of Munich Munich Germany

**Keywords:** computed tomography angiography, fibula free flap, lower limb vascular anatomy, perforator mapping, septocutaneous perforators

## Abstract

**Background:**

Reliable localization of septocutaneous fibular artery perforators is essential for fibula free flap planning. However, the influence of lower‐leg vascular anatomy on perforator number and spatial distribution remains unclear. This study aimed to analyze perforator number and spatial distribution using computed tomography angiography (CTA) with both fibula‐ and artery‐based reference systems.

**Methods:**

In this retrospective study, patients undergoing lower‐extremity CTA before mandibular continuity resection were screened. Parameters included run‐off status, Kim classification, vascular anomalies, atherosclerotic plaque, and morphometric characteristics of the fibula and fibular artery. Perforator number per limb and relative perforator position were assessed using fibula‐ and artery‐based reference systems. Associations were evaluated using nonparametric tests and multivariable regression models.

**Results:**

A total of 491 limbs from 247 patients were included, yielding 812 septocutaneous perforators. Limbs with fibular artery stenosis showed fewer perforators compared with limbs without stenosis (median 0.5 [0–2] vs. 2 [1–2], *p* < 0.001). Perforator distribution was nonuniform in both reference systems, with clustering in mid‐segments (*p* < 0.001). Notably, spatial distribution patterns differed depending on the reference system used. In multivariable analyses, non‐1A Kim run‐off classification was associated with a more proximal perforator position along the fibula (β = −10.0 percentage points, *p* < 0.001), whereas no limb‐level factor was associated with artery‐referenced perforator position, indicating greater stability of artery‐based mapping.

**Conclusion:**

Septocutaneous fibular artery perforators show reference‐dependent spatial variability. Fibular artery stenosis is associated with fewer perforators, whereas only Kim run‐off classification affects fibula‐referenced location. These findings support artery‐based mapping for fibula flap planning.

## 1 Introduction

The free fibula flap (FFF) is one of the most frequently used flaps in osseous reconstruction of the maxilla and mandible after ablative surgery (Okay et al. 2016; Ritschl et al. 2017; Ritschl, Kilbertus, et al. 2021; Ritschl, Mucke, et al. 2021). Its reliability is largely attributed to the consistent vascular anatomy of the fibular artery and its septocutaneous perforators, which supply the overlying skin paddle. Accurate identification and localization of these perforators can assist flap design, minimize donor‐site morbidity, and reduce intraoperative uncertainty (Allen et al. 2025; Lonie et al. 2016; Wallace et al. 2010).

Computed tomography angiography (CTA) has become an established modality for preoperative assessment of lower limb vascular anatomy (Garvey et al. 2012). Beyond evaluating atherosclerotic pathologies and detecting anatomical variants, CTA provides the possibility of mapping perforator location, course, and caliber (Alolabi et al. 2019; Golas et al. 2016). As a result, CTA‐based perforator mapping has increasingly replaced invasive angiography and intraoperative exploration as the primary planning tool for FFF harvest (Alolabi et al. 2022; Battaglia et al. 2017; Ettinger et al. 2018; Knitschke et al. 2021).

Beyond CTA, septocutaneous fibular artery perforators can be identified using several other techniques. High‐frequency color Doppler ultrasound allows preoperative localization and skin marking of perforators and can provide functional flow assessment (Gong et al. 2022; Miyamoto et al. 2022), while magnetic resonance angiography (MRA) offers radiation‐free visualization of perforator course and location within the posterolateral septum (Fukaya et al. 2010). Intraoperatively, handheld Doppler mapping can be used to confirm perforator signals and refine skin paddle design immediately prior to flap elevation (Jones et al. 1996; Yu et al. 2011).

The spatial distribution of fibular perforators has been described relative to bony landmarks, most commonly as a proportion of total fibular length (Iorio et al. 2012; Papadimas et al. 2009). This fibula‐based reference system is intuitive from a surgical perspective, as bony landmarks are readily identifiable intraoperatively (Schuderer et al. 2020; Yu et al. 2011).

The aim of this study was to perform a comprehensive CTA‐based analysis of septocutaneous fibular artery perforators, focusing on their anatomical distribution using both fibula‐ and artery‐based reference systems. By evaluating limb‐level vascular anatomy and perforator location, we sought to characterize spatial distribution patterns, assess factors associated with perforator occurrence, and clarify how the choice of reference system influences the interpretation of perforator anatomy.

## 2 Patients and Methods

### 2.1 Patient Selection and CTA Data Acquisition

All investigations were conducted in accordance with the principles of the Declaration of Helsinki. This retrospective imaging‐based study was approved by the Institutional Ethics Committee of the Technical University of Munich, Klinikum rechts der Isar (No.: 2023–459‐S‐SB). Due to the retrospective nature of the study and anonymization of all data, the requirement for informed consent was waived.

**FIGURE 1:**
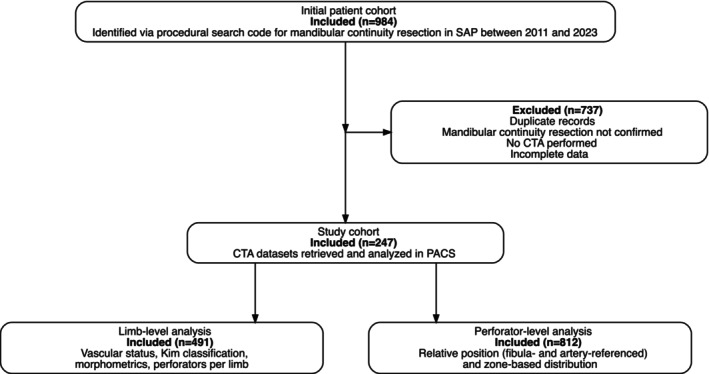
Flowchart illustrating patient selection, exclusion criteria, and derivation of limb‐ and perforator‐level analysis cohorts based on CTA data. Abbreviations: SAP,systems, applications and products in data processing; CTA, computed tomography angiography; PACS, picture archiving and communication system.

This retrospective cohort study included patients who underwent preoperative lower‐extremity CTA as part of routine planning for microvascular reconstruction following mandibular continuity resection between July 2012 and June 2023 at the Department of Oral and Maxillofacial Surgery, TUM University Hospital Klinikum Rechts der Isar, School of Medicine and Health, Technical University of Munich, Germany (Figure 1). Both lower limbs were analyzed independently. Limbs were excluded in cases of incomplete CTA datasets, insufficient image quality for reliable vascular assessment, or missing relevant imaging data. No exclusion based on vascular anatomy was applied.

Patient identification was performed using the institutional digital operating room database, applying the procedural code for mandibular continuity resection. CTA examinations were retrieved from the institutional picture archiving and communication system (PACS) and analyzed for both lower limbs separately. CTA examinations were performed using multidetector CT scanners with intravenous contrast administration and arterial phase acquisition. Images were reconstructed with a thin‐slice technique (slice thickness 0.9 mm) and analyzed using multiplanar reconstructions derived from axial source images.

All imaging‐derived measurements from CTA were pseudonymized prior to analysis and stored in a dedicated database for structured evaluation.

### 2.2 Radiological Assessment and CTA Measurements

**FIGURE 2:**
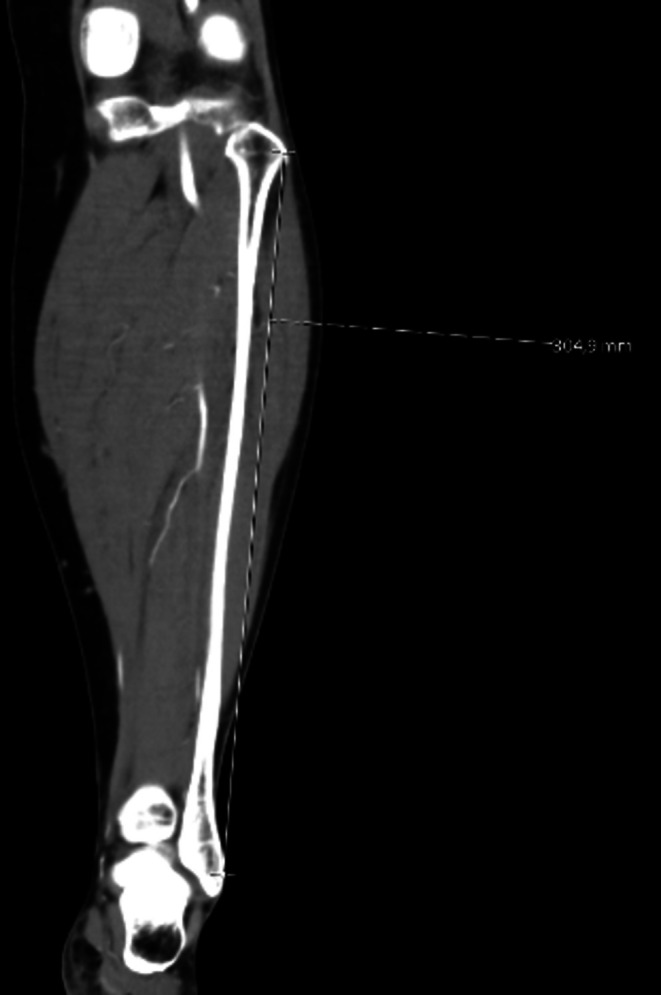
Coronal computed tomography angiography image of the lower leg illustrating fibular length measurement. The fibula length was measured in the coronal plane as the linear distance between the most lateral point of the fibular head (caput fibulae) and the most lateral point of the lateral malleolus.

**FIGURE 3:**
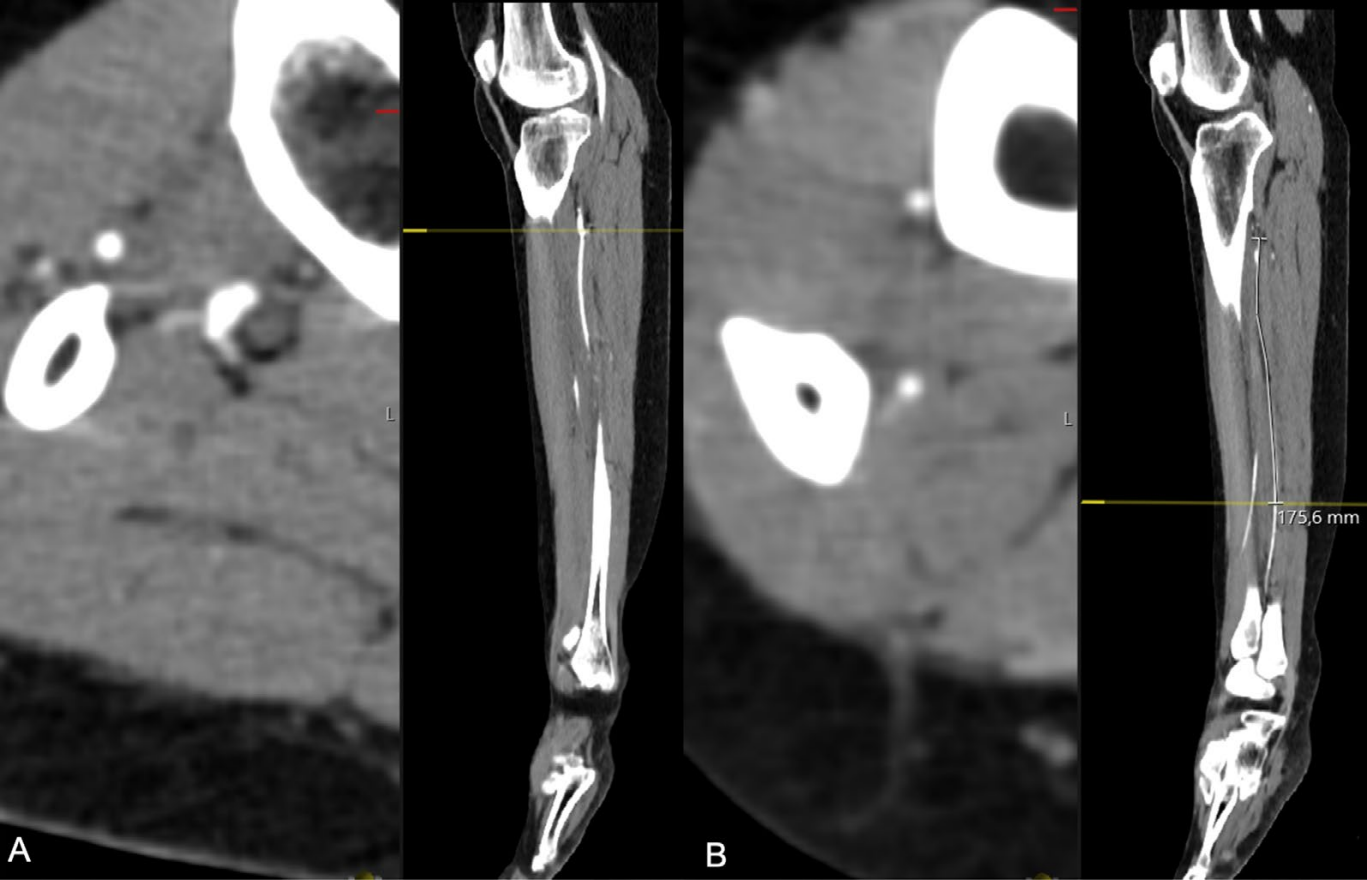
Axial and sagittal computed tomography angiography images illustrating fibular artery anatomy and perforator origin. In both panels, the axial plane is shown on the left and the corresponding sagittal plane on the right. (A) Axial and sagittal views at the level of bifurcation of the truncus tibiofibularis into the anterior tibial artery and the fibular artery. (B) Axial and sagittal views at the level of origin of a septocutaneous perforator arising from the fibular artery.

CTA datasets were analyzed using the institutional PACS viewer with multiplanar reconstruction enabled. For each limb, multiplanar reconstructions were generated in the axial, coronal, and sagittal planes to allow standardized and reproducible assessment of vascular anatomy and bony landmarks. In the coronal plane, overall vascular status was assessed, including evaluation of arterial patency, presence of stenosis, arterial aplasia, atherosclerotic plaque, and three‐vessel run‐off. Run‐off anatomy was classified according to the Kim classification system (Kim et al. 1989). Vascular findings were documented separately for right and left limbs. The total fibular length was measured as the linear distance between the most lateral point of the fibular head (*caput fibulae*) and the most lateral point of the lateral malleolus (Figure 2). This measurement served as the bony reference for subsequent relative perforator localization along the fibula. The tibiofibular trunk was identified in both the axial and sagittal planes. Its proximal origin and distal bifurcation were defined, and trunk length was measured as the linear distance between these two points. In addition, the truncus diameter was measured in the axial plane at its maximal caliber. The fibular artery was subsequently traced in the axial and sagittal planes along its entire course. The total length of the fibular artery was measured from its origin to its distal split. The distance between the fibular head and the origin of the fibular artery was recorded as an absolute measurement. Septocutaneous perforators arising from the fibular artery were identified primarily in the axial plane, where their origin from the main vessel could be visualized most reliably (Figure 3).

### 2.3 Limb‐ and Perforator‐Level Analyses

Analyses were performed at two anatomical levels. First, a limb‐based analysis was conducted, in which each lower limb represented an independent anatomical unit. At this level, global vascular characteristics were assessed, including overall arterial anatomy, Kim run‐off classification, presence of arterial aplasia, stenosis or atherosclerotic plaque, truncus tibiofibularis morphology, fibular artery length, and the total number of septocutaneous perforators per limb.

Second, a perforator‐based analysis was performed, in which each individual septocutaneous perforator represented a separate analytical unit. For each perforator, the axial level of origin was documented. Perforator positions were then referenced to both anatomical systems:

**Fibula‐based reference system:** Perforator location was expressed as a percentage of total fibular length, calculated from the fibular head (0%) to the lateral malleolus (100%).

**Artery‐based reference system:** Perforator location was expressed as a percentage of total fibular artery length, calculated from the arterial origin (0%) to the distal end of the artery (100%).

### 2.4 Statistical Analysis

Statistical analyses were performed using R (version 4.5.1). Continuous variables are reported as median with interquartile range (IQR), and categorical variables as absolute numbers and percentages. CTA‐derived parameters were analyzed on a limb‐level basis, with each limb considered an independent anatomical unit. Due to the retrospective design of the study, the availability of individual CTA‐derived variables varied between limbs; therefore, analyses were conducted using available‐case analysis, and variable‐specific denominators are reported where applicable. Associations between variables were assessed using appropriate parametric or nonparametric tests, with statistical significance defined as *p* < 0.05.

To assess the influence of limb‐level vascular and anatomical factors on septocutaneous perforator distribution, additional analyses were performed at the perforator level, with perforators nested within limbs. Perforator position was evaluated relative to both the fibular artery and the fibula, each expressed as a percentage of total length. Multivariable linear mixed‐effects models with a random intercept for limb were applied, including fibular artery stenosis, anomalies of the anterior and/or posterior tibial arteries, Kim runoff classification (1A vs. non‐1A), and the respective arterial or bony length as fixed effects. As the random‐intercept variance was negligible, results were confirmed using fixed‐effects linear regression models.

## 3 Results

### 3.1 Study Cohort and Limb‐Level Characteristics

A total of 491 lower limbs from 247 patients were included in the limb‐level analysis, comprising 246 right and 245 left limbs (Table 1). Three‐vessel run‐off was assessable in 485 limbs and was present in 423 cases (87.2%). Comparable rates were observed between right (88.1%) and left limbs (86.3%), while absence of three‐vessel run‐off was identified in 62 limbs (12.8%).

**TABLE 1:** Limb‐level vascular anatomy, run‐off patterns, and morphometric characteristics of the fibular artery assessed by computed tomography angiography.

Variable	Overall (*n* = 491)	Right limbs (*n* = 246)	Left limbs (*n* = 245)
Three‐vessel runoff			
Yes	423/485 (87.2%)	215/244 (88.1%)	208/241 (86.3%)
No	62/485 (12.8%)	29/244 (11.9%)	33/241 (13.7%)
Kim classification			
1A	402/432 (93.1%)	210/218 (96.3%)	192/214 (89.7%)
1B	9/432 (2.1%)	3/218 (1.4%)	6/214 (2.8%)
2A	7/432 (1.6%)	1/218 (0.5%)	6/214 (2.8%)
2B	5/432 (1.2%)	1/218 (0.5%)	4/214 (1.9%)
2C	4/432 (0.9%)	1/218 (0.5%)	3/214 (1.4%)
3B	5/432 (1.2%)	2/218 (0.9%)	3/214 (1.4%)
Vascular anomalies			
None	369 (75.1%)	206 (83.7%)	163 (66.5%)
Stenosis anterior or posterior tibial artery	39 (7.9%)	20 (8.1%)	19 (7.8%)
Stenosis both arteries	3 (0.6%)	2 (0.8%)	1 (0.4%)
Aplasia anterior or posterior tibial artery	54 (11.0%)	6 (2.4%)	48 (19.6%)
Aplasia both arteries	2 (0.4%)	1 (0.4%)	1 (0.4%)
Fibular artery stenosis	24 (4.9%)	11 (4.5%)	13 (5.3%)
Atherosclerotic plaque			
No	408 (83.1%)	185 (75.2%)	223 (91.0%)
Yes	83 (16.9%)	61 (24.8%)	22 (9.0%)
Fibula length (mm)	345.8 (326.7–361.9)	346.0 (326.4–361.9)	344.5 (326.6–362.5)
Distance caput fibulae → fibular artery origin (mm)	53.3 (45.3–63.0)	55.0 (47.0–65.2)	52.2 (44.0–61.0)
Fibular artery length (mm)	259.2 (241.0–279.7)	260.1 (241.1–278.5)	258.8 (240.1–281.0)
Truncus length (mm)	33.3 (24.0–41.7)	34.0 (26.3–42.0)	32.6 (23.0–40.8)
Truncus diameter (mm)	4.3 (3.8–4.9)	4.4 (3.8–5.0)	4.3 (3.9–4.9)
Number of perforators per limb	1 (1–2)	1 (1–2)	1 (1–2)

*Note:* Values are presented as *n* (%) or median with interquartile range (IQR). Vascular anatomy and morphometric measurements are reported for all evaluable limbs and stratified by side (right vs. left). Denominators vary according to the availability of specific computed tomography angiography (CTA)‐derived parameters.

Kim run‐off classification was available in 432 limbs. The majority of evaluable limbs were classified as Kim type 1A (93.1%). Less frequent patterns included type 1B (2.1%), type 2A (1.6%), type 2B (1.2%), type 2C (0.9%), and type 3B (1.2%). Non‐1A classifications were more frequently observed in left limbs than in right limbs.

No vascular anomalies were detected in 369 limbs (75.1%). Any vascular anomaly was identified in 122 limbs (24.8%), with a higher prevalence in left limbs (34.4%) compared with right limbs (15.0%). Atherosclerotic plaque was present in 83 limbs (16.9%) overall and was more common on the right side (24.8%) than on the left side (9.0%).

Median fibula length was 345.8 mm (326.7–361.9), with comparable measurements between right 346.0 mm (326.4–361.9) and left limbs 344.5 mm (326.6–362.5). The median distance from the fibular head to the origin of the fibular artery was 53.3 mm (45.3–63.0). Median fibular artery length measured 259.2 mm (241.0–279.7), while median tibiofibular trunk length was 33.3 mm (24.0–41.7). The median trunk diameter was 4.3 mm (3.8–4.9), with minimal side‐to‐side variation. The median number of septocutaneous perforators per limb was 1 (1–2) overall, with comparable values for right and left limbs.

### 3.2 Limb‐Level Factors Associated With Perforator Number

The presence of stenosis or aplasia of the anterior and/or posterior tibial artery was not significantly associated with the number of perforators per limb (*p* = 0.061, Table 2). In contrast, fibular artery stenosis was significantly associated with a reduced number of perforators (*p* < 0.001).

**TABLE 2:** Limb‐level parameters associated with the number of computed tomography angiography‐derived fibular artery septocutaneous perforators.

Parameter	*p*
Stenosis/aplasia posterior or anterior tibial artery (yes/no)	0.061
Fibular artery stenosis	**< 0.001**
Kim runoff classification	0.699
Fibula length (mm)	0.443
Tibiofibular trunk length (mm)	0.491
Fibular artery length (mm)	0.595
Distance caput fibulae → fibular artery origin (mm)	0.713

*Note:* Analysis restricted to limbs with a present fibular artery (fibular artery aplasia excluded); *n* = 480. Statistically significant results are shown in bold.

Limbs with fibular artery stenosis (*n* = 24, 4.9%) showed a markedly lower number of septocutaneous perforators compared with limbs without stenosis (median 0.5 [0–2] vs. 2 [1–2], *p* < 0.001). Fibular artery stenosis was significantly associated with the presence of atherosclerotic plaque (45.8% vs. 15.4%, *p* < 0.001). Patients with stenosis tended to be older than those without stenosis (median 67 [52–77] vs. 62 [54–69] years), although this difference did not reach statistical significance (*p* = 0.057).

No significant differences in perforator number were observed across Kim run‐off classifications (*p* = 0.699). Similarly, no significant associations were identified between perforator number and fibula length (*p* = 0.443), fibular artery length (*p* = 0.595), tibiofibular trunk length (*p* = 0.491), or the distance from the fibular head to the origin of the fibular artery (*p* = 0.713).

### 3.3 Perforator‐Level Anatomical Localization

A total of 812 septocutaneous perforators were included in the perforator‐level analysis. The median relative perforator position was 49.4% (30.3–63.2) along the fibula and 44.9% (19.3–63.5) along the fibular artery. The median absolute distance from the fibular head was 166.4 mm (101.8–212.6; Table 3).

**TABLE 3:** Relative and absolute anatomical position of septocutaneous fibular artery perforators.

Variable	Median (IQR)
Relative perforator height along fibula (%)	49.4 (30.3–63.2)
Relative perforator position along fibular artery (%)	44.9 (19.3–63.5)
Distance perforator to fibular head (mm)	166.4 (101.8–212.6)

*Note:* Values are reported as median (IQR). Relative perforator positions are expressed as percentages of total fibular length and total fibular artery length, respectively, while absolute distances are measured from the fibular head. Abbreviation: IQR, interquartile range.

### 3.4 Spatial Distribution of Perforators Along Arterial and Fibular Reference Systems

Perforator distribution along both reference systems was further analyzed by dividing the fibular artery and the fibula into 10 equal‐length zones (Table 4).

**TABLE 4:** Distribution of septocutaneous fibular artery perforators along arterial and fibular reference systems.

Arterial zone (% of length)	Perforators (*n*)	Percentage (%)
0–10	109	13.5
10–20	94	11.6
20–30	76	9.5
30–40	76	9.5
40–50	96	11.9
50–60	121	14.3
60–70	108	13.1
70–80	86	10.8
80–90	39	4.9
90–100	7	0.9
Total	812	100.0
		***p* < 0.001**

Fibular zone (% of length)	Perforators (*n*)	Percentage (%)
0–10	19	2.3
10–20	66	8.1
20–30	115	14.2
30–40	106	13.1
40–50	114	14.0
50–60	143	17.6
60–70	142	17.5
70–80	82	10.1
80–90	21	2.6
90–100	4	0.5
Total	812	100.0
		***p* < 0.001**

*Note:* Perforator locations are expressed as relative positions along the fibular artery and the fibula, respectively, each divided into 10 equal length zones. Values are presented as number (percentage). Chi‐square goodness‐of‐fit tests demonstrated a nonuniform distribution of perforators across zones for both arterial and fibular reference systems (*p* < 0.001 for both). Statistically significant results are shown in bold.

**FIGURE 4:**
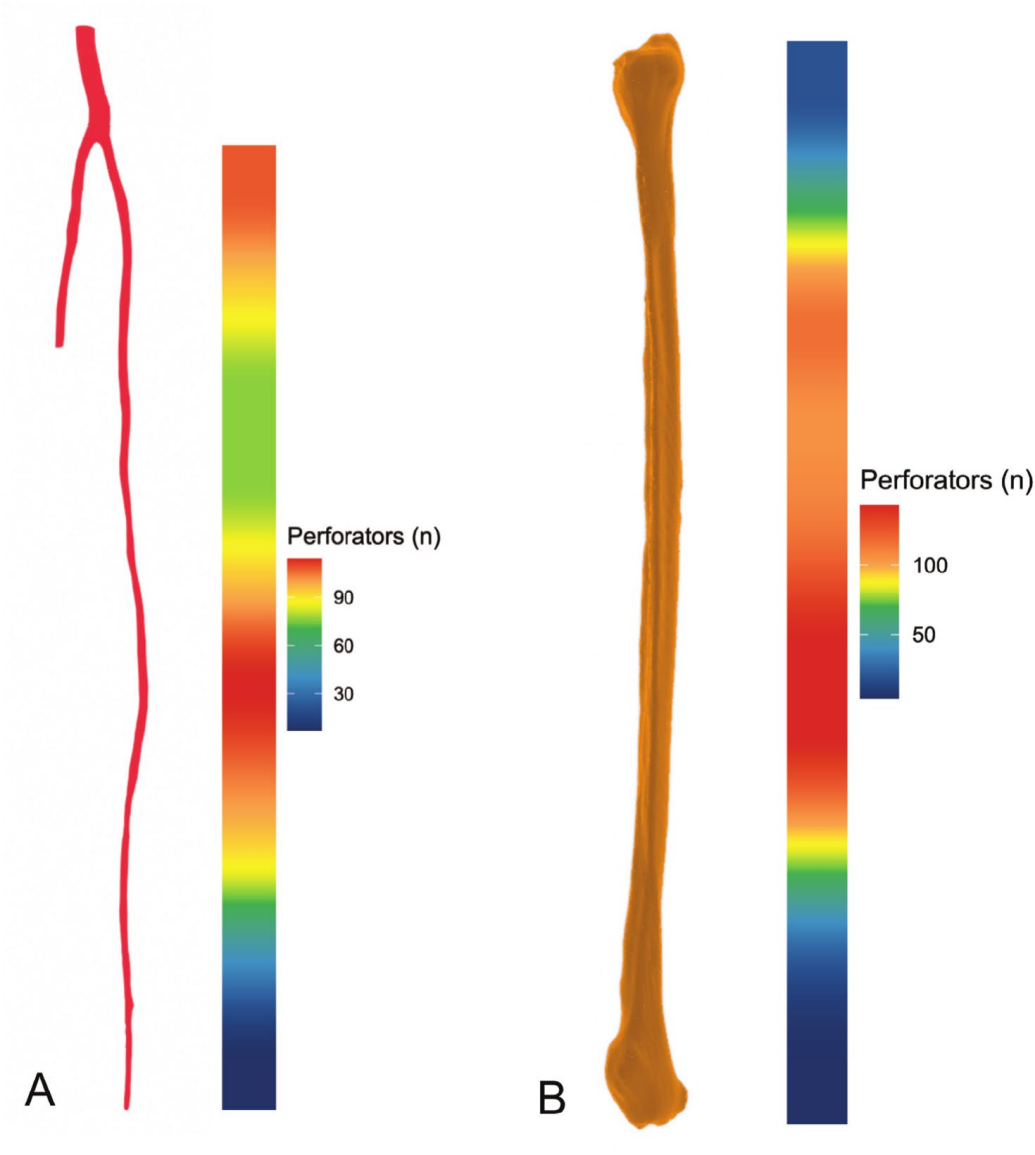
Arterial‐ (A) and fibula‐based (B) heatmap representation of septocutaneous fibular artery perforator distribution displayed alongside schematic representations of the fibular artery and fibula. Color intensity reflects the relative frequency of perforator occurrence, with higher intensity indicating greater perforator density and clustering within each segment.

Along the arterial axis, perforators were unevenly distributed across zones (*χ* ^2^ goodness‐of‐fit test, *p* < 0.001, Figure 4). The heatmap color scale represents the relative frequency of perforator occurrence, with increasing color intensity indicating higher perforator density. Accordingly, areas of greater intensity reflect regions of greater perforator clustering. The highest frequencies were observed in the proximal 0%–10% zone (13.5%) and in the mid‐arterial segments between 50%–60% (14.3%) and 60%–70% (13.1%). In contrast, perforators were infrequent in the distal arterial segments, with only 4.9% located between 80% and 90% and 0.9% between 90% and 100% of arterial length.

In contrast, fibula‐based mapping demonstrated a different distribution pattern, which was likewise nonuniform (*χ* ^2^ goodness‐of‐fit test, *p* < 0.001). Perforators clustered predominantly in the mid‐fibular region, with the highest frequencies between 50%–60% (17.6%) and 60%–70% (17.5%) of fibular length, followed by the 20%–30% (14.2%) and 40%–50% (14.0%) zones. Proximal (0%–10%) and distal (90%–100%) fibular segments contained only a small proportion of perforators.

### 3.5 Association Between Limb‐Level Variables and Perforator Distribution

In multivariate analysis assessing the relative position of septocutaneous perforators along the fibular artery, none of the investigated limb‐level variables were significantly associated with perforator location (Table 5). Neither fibular artery stenosis, anomalies of the anterior or posterior tibial arteries, and Kim runoff classification, nor fibular artery length demonstrated a significant effect on artery‐referenced perforator position (all *p* > 0.100).

**TABLE 5:** Multivariate analysis of limb‐level factors associated with the relative position of septocutaneous perforators of the fibular artery.

Outcome (reference system)	Predictor	β (estimate)	Standard‐error	*p*
Relative perforator position along fibular artery (%)	Fibular artery stenosis (yes vs. no)	+1.80	6.78	0.791
	Anomaly anterior or posterior tibial artery (yes vs. no)	+3.27	2.83	0.249
	Kim non‐1A classification	−5.22	3.56	0.143
	Fibular artery length (per mm)	−0.04	0.03	0.115
Relative perforator position along fibula (%)	Fibular artery stenosis (yes vs. no)	−0.42	5.27	0.937
	Anomaly anterior or posterior tibial artery (yes vs. no)	+1.42	2.22	0.524
	Kim non‐1A classification	−10.01	2.79	**< 0.001**
	Fibula length (per mm)	−0.04	0.03	0.088

*Note:* Perforator position was assessed relative to the fibular artery and the fibula, expressed as percentages of total length. Linear mixed‐effects models with a random intercept for limb were applied; results were confirmed using fixed‐effects linear regression due to a singular random‐effects structure. Regression coefficients (β), standard errors, and *p* values are shown. Statistically significant results are shown in bold.

In contrast, when perforator position was referenced to fibular length, a distinct association emerged. A non‐1A Kim runoff classification was independently associated with a significantly altered perforator distribution along the fibula, corresponding to a mean shift of approximately 10 percentage points compared with Kim 1A anatomy (β = −10.0, *p* < 0.001). Fibula length showed a nonsignificant trend toward a more proximal perforator position (*p* = 0.088), while fibular artery stenosis and anomalies of the remaining lower‐leg arteries were not associated with perforator location.

Random intercept variance for limb was estimated at zero in both models, indicating minimal additional clustering effects at the limb level. Accordingly, fixed‐effects linear regression models yielded identical results.

## 4 Discussion

This CTA‐based study provides a comprehensive limb‐ and perforator‐level analysis of septocutaneous fibular artery perforators and yields several clinically and anatomically relevant findings. Importantly, the novelty of this study lies not in the description of perforators per se, but in the systematic comparison of artery‐ and fibula‐based reference systems. The key results can be summarized as follows: (1) the number of septocutaneous perforators visible in CTA per limb is largely independent of lower‐leg run‐off anatomy and morphometric parameters, with the notable exception of fibular artery stenosis; (2) perforator distribution is strongly reference‐dependent and differs substantially between artery‐ and fibula‐based systems; and (3) limb‐level vascular anatomy, specifically Kim runoff classification, influences perforator position only when a bone‐based reference system is applied.

### 4.1 Limb‐Level Analysis

At the limb‐level, most lower limbs demonstrated preserved three‐vessel runoff and Kim type 1A anatomy, reflecting a predominantly standard vascular configuration in line with observations made in other studies (Abou‐Foul and Borumandi 2016; Abou‐Foul et al. 2017; Kim et al. 2019). Although vascular anomalies of the anterior or posterior tibial arteries were relatively common, they were not associated with the number of septocutaneous perforators. In contrast, fibular artery stenosis was significantly associated with a reduced radiological perforator count, underscoring the dependence of septocutaneous perforators on adequate inflow within the fibular artery itself. Although patients with fibular artery stenosis tended to be older, age was not significantly associated with stenosis in our cohort, suggesting that the observed reduction in perforator number is unlikely to be solely explained by age‐related vascular changes. In addition, atherosclerotic plaque was significantly associated with fibular artery stenosis, indicating that underlying vascular disease may contribute to the observed relationship. Although previous studies have not reported an association between fibular artery stenosis and perforator number (Knitschke et al. 2021; Schuderer et al. 2020), our findings align with the concept that perforator presence is primarily governed by the integrity of the source vessel rather than by collateral or compensatory changes in adjacent arteries (Fukaya et al. 2010; Kumar et al. 2023; Papadimas et al. 2009). Although fibular artery stenosis was associated with a reduced number of perforators, it was not associated with perforator position in multivariable analysis, indicating that the reduced perforator count does not substantially alter the overall spatial distribution pattern.

### 4.2 Perforator‐Level Analysis

Perforator‐level analyses in the present study demonstrated differences in spatial distribution depending on the anatomical reference system used. We particularly emphasize the value of our heatmap analysis, which provided a clear and intuitively interpretable visual representation of perforator distribution. By projecting the data onto two distinct reference systems, the vessel‐based axis of the fibular artery and the anatomy‐based longitudinal axis of the fibula, we were able to demonstrate systematically different distribution patterns. The heatmap not only confirmed a significantly nonuniform distribution of perforators within both systems (*p* < 0.001), but also made the discrepancies between vascular and osseous referencing immediately apparent. Along the arterial axis, perforators predominated in the proximal and mid‐arterial segments, whereas distal segments were markedly underrepresented. In contrast, fibula‐based mapping demonstrated a pronounced clustering within the mid‐fibular region. This side‐by‐side visual comparison underscores that the choice of reference system substantially influences the perceived spatial distribution of perforators and may therefore have direct implications for preoperative planning and intraoperative orientation in fibula flap surgery. The relevance of reference‐system choice is also supported by prior literature. Iorio et al. (2012) demonstrated a clear clustering of peroneal artery perforators around the 0.6 interval of fibular length using bone‐based indexing, whereas recent CTA‐based analysis by Zhao et al. (2025) showed that perforator distribution and perceived clustering vary when evaluated relative to the fibular artery origin versus along the fibula. Classical anatomical and clinical studies have consistently described septocutaneous perforators as being most frequently located at the beginning of the distal third or at the junction of the middle and distal thirds of the fibula when referenced to bony landmarks, supporting bone‐based mapping as a practical and reproducible approach for flap planning (Cho et al. 2001; Jones et al. 1996; Papadimas et al. 2009; Poulet et al. 2022; Yu et al. 2011). Together, these findings support that reported perforator “hotspots” are inherently reference‐dependent.

Our results demonstrate that artery‐ and bone‐based reference systems describe perforator location from fundamentally different perspectives. Artery‐based mapping reflects the intrinsic branching pattern of septocutaneous perforators along the fibular artery and shows a relatively stable distribution, largely independent of limb‐level vascular variation (Abou‐Foul et al. 2017).

In contrast, bone‐based mapping describes perforator location in relation to the fibula itself and therefore incorporates both vascular anatomy and limb morphology. As a result, bone‐referenced perforator positions varied with differences in lower‐leg vascular configuration, particularly in limbs with non‐1A Kim runoff patterns (Ongsiriporn et al. 2021; Poulet et al. 2022). These findings indicate that the two reference systems are not interchangeable: artery‐based mapping captures vascular branching stability, whereas bone‐based mapping reflects the clinically relevant relationship between vessels and surgical landmarks.

Consistent with prior cadaveric, clinical, and imaging‐based studies, bone‐based reference systems using the fibular head and lateral malleolus remain highly relevant for surgical practice, given their intraoperative visibility and reproducibility (Ettinger et al. 2018, 2022; Papadimas et al. 2009). Advanced imaging modalities such as CTA and MRA further enhance the accuracy of bone‐based perforator localization, achieving subcentimeter precision and high sensitivity for septocutaneous perforator detection (Battaglia et al. 2017; Cirelli Jr. et al. 2025; Garvey et al. 2012; Schuderer et al. 2020). Our findings complement this body of literature by demonstrating that artery‐based and bone‐based mapping are not interchangeable and that discrepancies between the two reflect true anatomical differences rather than methodological error.

### 4.3 Clinical Translation

From a surgical perspective, this distinction is highly relevant for preoperative planning of free fibula or perforator flaps. In FFF harvesting, the precise location of septocutaneous perforators relative to bony landmarks directly influences segmental osteotomy design, selection of skin paddle position, and the feasibility of multisegment reconstructions (Battaglia et al. 2017; Ettinger et al. 2022). When multiple osteotomies are required, preservation of at least one reliable perforator within the planned skin‐bearing segment is essential. Bone‐based mapping therefore provides critical information for determining safe osteotomy intervals, whereas artery‐based mapping enhances understanding of intrinsic vascular branching stability (Ettinger et al. 2018). Integrating both reference systems may optimize flap design with skin paddle positioning, particularly in complex mandibular reconstructions requiring tailored bone segment geometry. For example, in complex mandibular reconstructions requiring multiple osteotomies, reliance solely on bone‐based landmark estimates may result in a mismatch between planned osteotomy sites and the actual location of septocutaneous perforators. This may necessitate intraoperative modification of the skin paddle or compromise flap perfusion. Incorporating artery‐based CTA mapping into preoperative planning may therefore improve surgical predictability, facilitate perforator‐preserving osteotomies, and enhance flap safety.

Importantly, our findings do not contradict the well‐documented interindividual variability of septocutaneous perforators reported in the literature. Previous studies consistently demonstrate variability in perforator number, caliber, and exact location, particularly in relation to global limb vascular anatomy (Holzle et al. 2011; Iorio et al. 2012; Ribuffo et al. 2010). Rather, our results refine this concept by demonstrating that variability depends strongly on the chosen anatomical reference system as well as radiological visibility versus intraoperative exposure. While global vascular variations influence the number of perforators and their spatial relationship to the fibula, the relative branching pattern along the fibular artery itself appears comparatively stable once a patent artery is present.

Several limitations should be acknowledged. The retrospective design and CTA‐based assessment limit functional evaluation of perforator caliber and flow. Additionally, most limbs harbored a small number of septocutaneous perforators, resulting in minimal variance and a singular random‐effects structure in mixed‐effects models. However, confirmation using fixed‐effects linear regression yielded identical results, supporting the robustness of the findings.

## 5 Conclusions

These findings support artery‐based CTA mapping as an objective, stable, and anatomically consistent approach for perforator localization in fibula free flap planning, particularly in the presence of vascular variants. Our analysis demonstrates that perforator distribution differs substantially depending on whether a vessel‐ or fibula‐based reference system is applied, with significant clustering patterns becoming evident through heatmap visualization.

Importantly, the integration of perforator localization into CAD/CAM‐based planning workflows represents a critical next step. Incorporating reliable, reference‐standardized perforator mapping into virtual surgical planning could enhance flap design, optimize skin paddle positioning, and improve the alignment between reconstructive requirements and vascular anatomy. Such integration may ultimately increase surgical precision, reduce intraoperative uncertainty, and contribute to more predictable outcomes in FFF reconstruction.

## Author Contributions

All authors had full access to all the data in the study and had final responsibility for the decision to submit for publication. At least two authors (Jannik Ketschau and Lucas M. Ritschl) assessed and verified the underlying data.

## Funding

The authors have nothing to report.

## Ethics Statement

The study was conducted in accordance with the Declaration of Helsinki. Approval was obtained from the institutional ethics committee prior to data collection (No.: 2023–459‐S‐SB). Due to the retrospective nature of the study and the use of anonymized data, the requirement for informed consent was waived.

## Conflicts of Interest

The authors declare no conflicts of interest.

## Data Availability

The datasets generated during and analyzed during the current study are available from the corresponding author on reasonable request.
